# A database of the healthy human spinal cord morphometry in the PAM50 template space

**DOI:** 10.1162/imag_a_00075

**Published:** 2024-02-02

**Authors:** Jan Valošek, Sandrine Bédard, Miloš Keřkovský, Tomáš Rohan, Julien Cohen-Adad

**Affiliations:** NeuroPoly Lab, Institute of Biomedical Engineering, Polytechnique Montreal, Montreal, Canada; Mila - Quebec AI Institute, Montreal, Canada; Department of Neurosurgery, Faculty of Medicine and Dentistry, Palacký University Olomouc, Olomouc, Czechia; Department of Neurology, Faculty of Medicine and Dentistry, Palacký University Olomouc, Olomouc, Czechia; Department of Radiology and Nuclear Medicine, University Hospital Brno and Masaryk University, Brno, Czechia; Functional Neuroimaging Unit, CRIUGM, Université de Montréal, Montreal, Canada; Centre de Recherche du CHU Sainte-Justine, Université de Montréal, Montreal, Canada

**Keywords:** Spinal cord, morphometric measures, normalization, normative values

## Abstract

Measures of spinal cord morphometry computed from magnetic resonance images serve as relevant prognostic biomarkers for a range of spinal cord pathologies, including traumatic and non-traumatic spinal cord injury and neurodegenerative diseases. However, interpreting these imaging biomarkers is difficult due to considerable intra- and inter-subject variability. Yet, there is no clear consensus on a normalization method that would help reduce this variability and more insights into the distribution of these morphometrics are needed. In this study, we computed a database of normative values for six commonly used measures of spinal cord morphometry: cross-sectional area, anteroposterior diameter, transverse diameter, compression ratio, eccentricity, and solidity. Normative values were computed from a large open-access dataset of healthy adult volunteers (N = 203) and were brought to the common space of the PAM50 spinal cord template using a newly proposed normalization method based on linear interpolation. Compared to traditional image-based registration, the proposed normalization approach does not involve image transformations and, therefore, does not introduce distortions of spinal cord anatomy. This is a crucial consideration in preserving the integrity of the spinal cord anatomy in conditions such as spinal cord injury. This new morphometric database allows researchers to normalize based on sex and age, thereby minimizing inter-subject variability associated with demographic and biological factors. The proposed methodology is open-source and accessible through the Spinal Cord Toolbox (SCT) v6.0 and higher.

## 1 Introduction

### 1.1 Spinal cord morphometry measures

The spinal cord plays a vital role in the central nervous system by transmitting sensory and motor signals between the brain and the rest of the body. It also contains essential networks responsible for functions such as locomotion and pain processing. Structural magnetic resonance imaging (MRI) is commonly used to assess spinal cord macrostructure and to compute measures of spinal cord morphometry like cross-sectional area (CSA) or anteroposterior (AP) diameter. The morphometric measures serve as objective indicators to evaluate spinal cord pathologies, such as the extent of spinal cord atrophy in multiple sclerosis (Losseff et al., 1996; Mina et al., 2021; Rocca et al., 2019) and amyotrophic lateral sclerosis (El Mendili et al., 2023; Paquin et al., 2018) or the severity of spinal cord injury and spinal cord compression in traumatic and non-traumatic spinal cord injury, respectively (Badhiwala et al., 2020; David et al., 2019; Miyanji et al., 2007).

However, interpreting morphometric measures is challenging due to considerable inter-subject variability associated with demographic and biological factors. For example, significantly smaller CSA is consistently reported in females relative to males (Bédard & Cohen-Adad, 2022; Engl et al., 2013; Mina et al., 2021; Papinutto et al., 2015, 2020; Rashid et al., 2006; Solstrand Dahlberg, Viessmann, & Linnman, 2020; Yanase et al., 2006). Similarly, studies showed an association of spinal cord CSA with cervical cord length (Martin et al., 2017a, 2017b; Oh et al., 2014), spinal canal area, and spinal canal diameters (Kesenheimer et al., 2021; Papinutto et al., 2020). Other factors, such as brain volume, intracranial volume, and thalamic volume also showed a strong correlation with spinal cord CSA (Bédard & Cohen-Adad, 2022; Papinutto et al., 2015, 2020; Rashid et al., 2006; Solstrand Dahlberg, Viessmann, & Linnman, 2020).

As for weight and height, studies showed only a moderate correlation with spinal cord CSA (Papinutto et al., 2020; Yanase et al., 2006) or did not show any significant association (Bédard & Cohen-Adad, 2022; Papinutto et al., 2020; Solstrand Dahlberg, Viessmann, & Linnman, 2020). Likewise, only a weak non-significant association was reported between spinal cord CSA and age (Bédard & Cohen-Adad, 2022; Kato et al., 2012; Papinutto et al., 2020; Yanase et al., 2006). A single study with a wide cohort age range reported that CSA increases until about 45 years of age and then begins to decrease (Papinutto et al., 2020).

In addition to inter-subject variability, spinal cord anatomy varies depending on the level. In agreement with anatomical textbooks (Standring, 2020), studies have shown an increase in CSA around vertebral levels C4-C5 corresponding to cervical enlargement (De Leener et al., 2018; Frostell et al., 2016; Horáková et al., 2022; Martin et al., 2017b; Mina et al., 2021; Rocca et al., 2019).

### 1.2 Normalization strategies

Various normalization strategies have been proposed to account for the above-mentioned factors on spinal cord morphometric measures. Sex was used for CSA normalization in several works (Bédard & Cohen-Adad, 2022; Kesenheimer et al., 2021; Papinutto et al., 2020; Rashid et al., 2006). Other studies proposed spinal cord length as a normalization factor (El Mendili et al., 2023; Martin et al., 2017a, 2017b; Oh et al., 2014; Rocca et al., 2019). Additionally, combining spinal cord length with a Z-score normalization was proposed to account for variations along the superior–inferior axis (Martin et al., 2017a, 2017b). Another approach taking into account the dependency of spinal cord anatomy on a level involved the normalization of morphometric measures from the compression site using non-compressed levels above and below (Guo et al., 2022; Miyanji et al., 2007). Finally, several studies normalized CSA using the spinal canal and brain metrics, including spinal canal area, spinal canal diameter, brain volume, intracranial volume, thalamic volume, and head size normalization factor (Bédard & Cohen-Adad, 2022; Horsfield et al., 2010; Kesenheimer et al., 2021; Papinutto et al., 2020; Rashid et al., 2006; Rocca et al., 2019).

While normalization strategies showed promising outcomes, there is currently no accepted consensus on which method to use (Cohen-Adad et al., 2021b; Papinutto et al., 2020), and their implementation is challenging. First, measuring the spinal cord length and the spinal canal area can be time-consuming if done manually. Second, obtaining brain MRI scans, necessary for assessing brain and thalamic volumes, may not be routinely available in spinal cord MRI protocols, and neurodegenerative diseases such as multiple sclerosis can influence brain measurements and potentially introduce bias during normalization.

### 1.3 Spinal cord template

Similarly to brain studies, spinal cord research involving multiple subjects frequently relies on templates—standardized, high-resolution images of the human spinal cord used as a reference for comparing and analyzing individual spinal cord scans. A commonly used spinal cord template is the PAM50 (De Leener et al., 2018). The process of aligning individual single-subject images to the template typically involves a series of non-linear image transformations, which may introduce inaccuracies when computing morphometric measures in the PAM50 vs. in the native subject’s space. This is an important consideration, especially in subjects with altered spinal cord anatomy, such as patients with spinal cord injury.

### 1.4 Normative values

Several multi-subject studies have provided normative values for spinal cord morphometry (Frostell et al., 2016; Horáková et al., 2022; Kato et al., 2012; De Leener et al., 2018; Taso et al., 2016). However, these studies show inconsistency in their reporting. Some authors only provided values for intervertebral discs (De Leener et al., 2018; Horáková et al., 2022), while others presented values averaged across multiple vertebral levels (Taso et al., 2016). Notably, none of these studies have presented normative values separated by sex.

### 1.5 Study objective

In this study, we present a database of healthy normative values for six commonly used measures of spinal cord morphometry built using a new fully automatic normalization approach. The interactive database is available online (https://preprint.neurolibre.org/10.55458/neurolibre.00017/) and allows filtering by sex, age, and MRI vendors. The proposed methodology is open-source and accessible through the Spinal Cord Toolbox (SCT) (De Leener et al., 2017), and can be used in future multi-subject studies to minimize inter- and intra-subject variability.

## 2 Materials and Methods

### 2.1 Data and participants

We used data from the *spine-generic* multi-subject dataset (Cohen-Adad et al., 2021b). The dataset is open-access, organized according to the Brain Imaging Data Structure (BIDS) standard (Gorgolewski et al., 2016; Karakuzu et al., 2022) and managed using git-annex (https://git-annex.branchable.com) in this GitHub repository: https://github.com/spine-generic/data-multi-subject/tree/r20230223.

Adult healthy participants were scanned across 43 centers on 3 T MRI scanners from 3 vendors (GE: N = 28, Philips: N = 36, and Siemens: N = 139) using the consensus *spine-generic* acquisition protocol (Cohen-Adad et al., 2021a). We used the 3D sagittal T2-weighted (T2-w) images (0.8 mm^3^) acquired using the SPACE (Siemens), VISTA (Philips), or CUBE (GE) sequences. We chose the T2-w over the 1 mm isotropic T1-weighted (T1-w) because (i) the former sequence is less sensitive to subject motion and (ii) the spatial resolution is better (0.8 mm isotropic), providing more precise morphometric measures. For additional considerations, see Discussion section 4.5. For more details on sequence parameters, see Cohen-Adad et al. (2021a, 2021b).

Two experienced radiologists (M.K. and T.R.) evaluated MRI scans with a focus on the presence of spinal cord compression. Spinal cord compression was defined as a change in spinal cord contour at the level of an intervertebral disc on an axial or sagittal MRI plane compared with that at the midpoint level of neighbouring vertebrae (Kadanka et al., 2017; Keřkovský et al., 2017). Minor abnormalities such as mild disc protrusions, spine misalignment, or minimal widening of the spinal cord central canal were not considered significant pathologies.

Qualitative assessment of the *spine-generic* dataset by two experienced radiologists revealed mostly mild spinal cord compression in 64 out of the total 267 volunteers (see the “pathology” column in this spreadsheet: https://github.com/spine-generic/data-multi-subject/blob/r20230223/participants.tsv). Those volunteers were excluded from the further analysis. The final cohort used for the normative database construction consisted of 203 healthy subjects (105 males and 98 females). Detailed demographic characteristics are provided in Table 1.

**Table 1.** The demographic characteristics of subjects included in the normative database.

	Whole cohort	Males	Females	p-Value Males-Females
Number of subjects	203	105	98	-
Age [y.o.]	28.7 ± 5.6	29.0 ± 5.6	28.3 ± 5.6	.241
Height [cm]	172.5 ± 9.9	179.3 ± 7.7	165.4 ± 6.1	<.001
Weight [kg]	67.7 ± 13.4	75.9 ± 10.8	58.9 ± 9.9	<.001

The values are shown as mean and standard deviation.

### 2.2 Data pre-processing

**Fig. 1.**
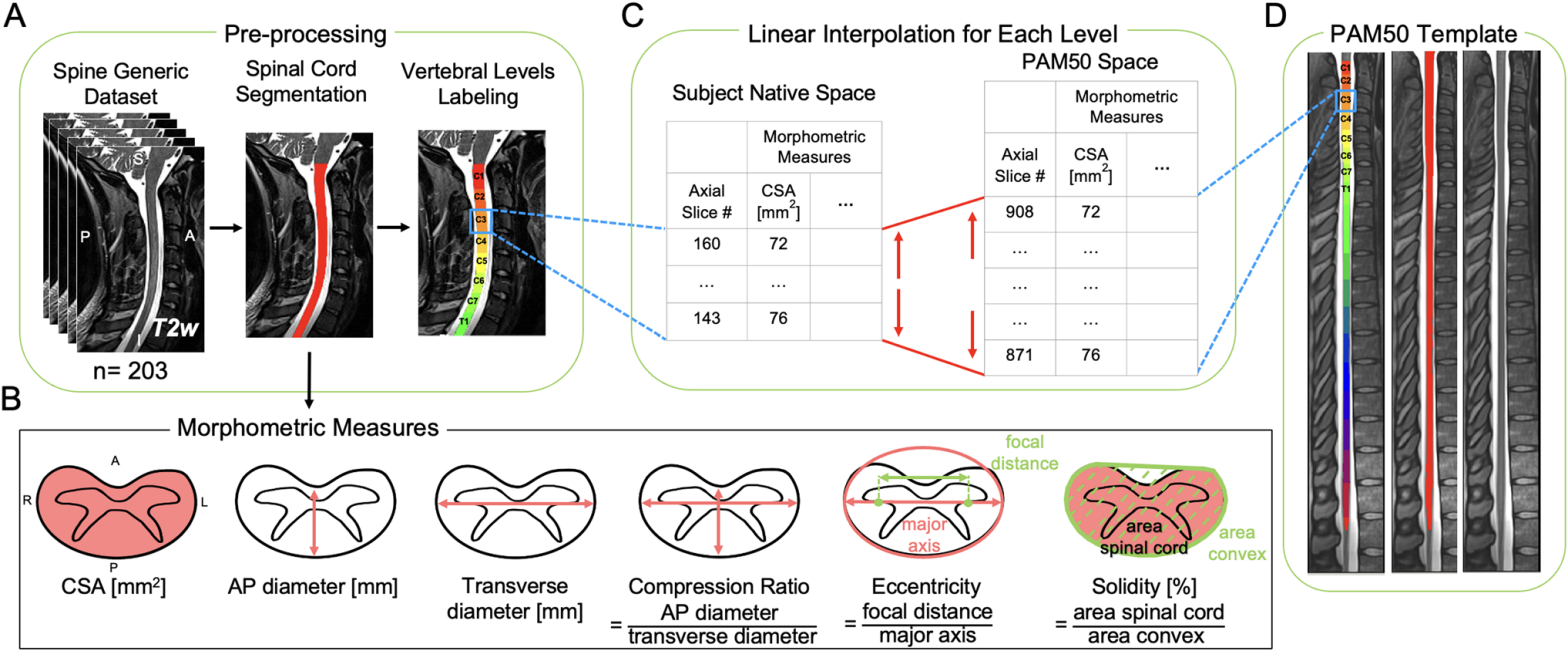
Schematic representation of the normalization approach. (A) T2-weighted images of 203 participants from the spine-generic dataset (multi-subject) were used. The spinal cord was segmented, and vertebral levels were identified automatically using the Spinal Cord Toolbox (SCT). (B) Six morphometric measures were computed for each axial slice from the single-subject segmentation masks. (C) For each vertebral level, the number of slices in the subject native space and the corresponding vertebral level in the PAM50 template (D) were identified. Then, the morphometric measures were linearly interpolated to the PAM50 space using the number of slices in the PAM50 template and the subject native space for each vertebral level.

The image processing was performed automatically using SCT v6.0 (De Leener et al., 2017). For each participant, the spinal cord was segmented using a deep learning-based algorithm (Gros et al., 2019) and the intervertebral discs were labeled (Ullmann et al., 2014) to generate the cord segmentation labeled with vertebral levels (Fig. 1A).

The spinal cord segmentation and disc labels were visually inspected using SCT’s quality control report (*sct_qc* function: https://spinalcordtoolbox.com/user_section/command-line.html#sct-qc) and manually corrected when necessary by J.V. and S.B. The manual corrections ensured that the spinal cord segmentation masks used for the computation of morphometric measures were reliable. Segmentation masks were corrected using FSLeyes image viewer (McCarthy, 2022) by adding or removing voxels when appropriate. Regarding vertebral labeling corrections, we manually identified the posterior tip of the intervertebral discs using SCT’s *sct_label_utils* function (De Leener et al., 2017) when it was necessary.

### 2.3 Normalization

Figure 1 shows a schematic representation of the normalization approach based on linear interpolation of morphometric measures from the subject’s native space to the anatomical dimensions of the PAM50 spinal cord template (De Leener et al., 2018).

After pre-processing (i.e., spinal cord segmentation and labeling), the morphometric measures were computed across individual axial slices from the spinal cord segmentation mask in the subject’s native space considering the angulation of the spinal cord (i.e., the angle between the spinal cord centerline and the axial plane). Then, the number of axial slices corresponding to each vertebral level was identified in both the subject’s native space and in the PAM50 template based on the labeled segmentation. Finally, the computed morphometric measures were linearly interpolated to the PAM50 anatomical dimensions based on the number of slices for each vertebral level in the native space and the PAM50 template.

The following morphometric measures were computed using SCT’s *sct_process_segmentation* for each participant: cross-sectional area (CSA), anteroposterior (AP) diameter, transverse diameter, compression ratio, eccentricity, and solidity (Fig. 1B).

Spinal cord CSA reflects the atrophy of the spinal cord and is computed as the area of the spinal cord in the transverse plane. The AP diameter is the measurement of the diameter of the spinal cord in the anterior–posterior direction, while the transverse diameter is the measurement of the diameter of the spinal cord from side to side. The compression ratio reflects the flattening of the spinal cord and is defined as the ratio of the AP diameter and the transverse diameter. Eccentricity is calculated by dividing the distance between the two focal points of the ellipse by the length of its longest diameter (the major axis). The value is in the interval [0, 1]. A lower eccentricity value (closer to 0) suggests a more circular spinal cord cross-section. Solidity is used to measure the indentation of the spinal cord and is defined as the ratio of the area representing the spinal cord to the area of the smallest convex polygon surrounding all positive pixels in the image. Solidity is relevant in detecting non-convex shapes, for instance, in subjects with spinal cord compression.

### 2.4 Normative values and interactive database

The normative values were calculated as mean and standard deviation across participants for slices in PAM50 space corresponding to each intervertebral disc and the middle of each vertebral level. The normative values are provided for the whole cohort and separated by sex.

For convenient introspection of the morphometric measures, interactive figures were created using the Plotly (https://github.com/plotly/plotly.py) Python library v5.9.0. The figures allow interactive visualization of normative values for any slice in the PAM50 space and filtering for sex, age decades, and MRI vendors. The figures show values per slice (instead of per vertebral level), to prevent the loss of information that would arise if values were averaged within each vertebral level.

### 2.5 Statistical analysis

Statistical analysis was conducted using the SciPy Python library v1.10.1 (Virtanen et al., 2020). Descriptive statistics, including mean and standard deviation, were computed for age, height, and weight. The Shapiro–Wilk normality test was used to assess data normality. Differences between males and females in age, height, and weight were examined using the Wilcoxon rank-sum test. Morphometric measures in PAM50 space were averaged across participants for each slice and compared between sex and MRI vendors using the Wilcoxon rank-sum test. The significance level was set to alpha = 0.001.

The inter-subject coefficient of variation (COV), defined as the ratio of standard deviation and mean, was computed per slice for all morphometrics measures. The COV was then averaged for individual vertebral levels. Additionally, the mean COV for the whole cervical spinal cord was computed as average across all slices.

## 3 Results

### 3.1 Morphometric measures - variation across vertebral levels

**Fig. 2.**
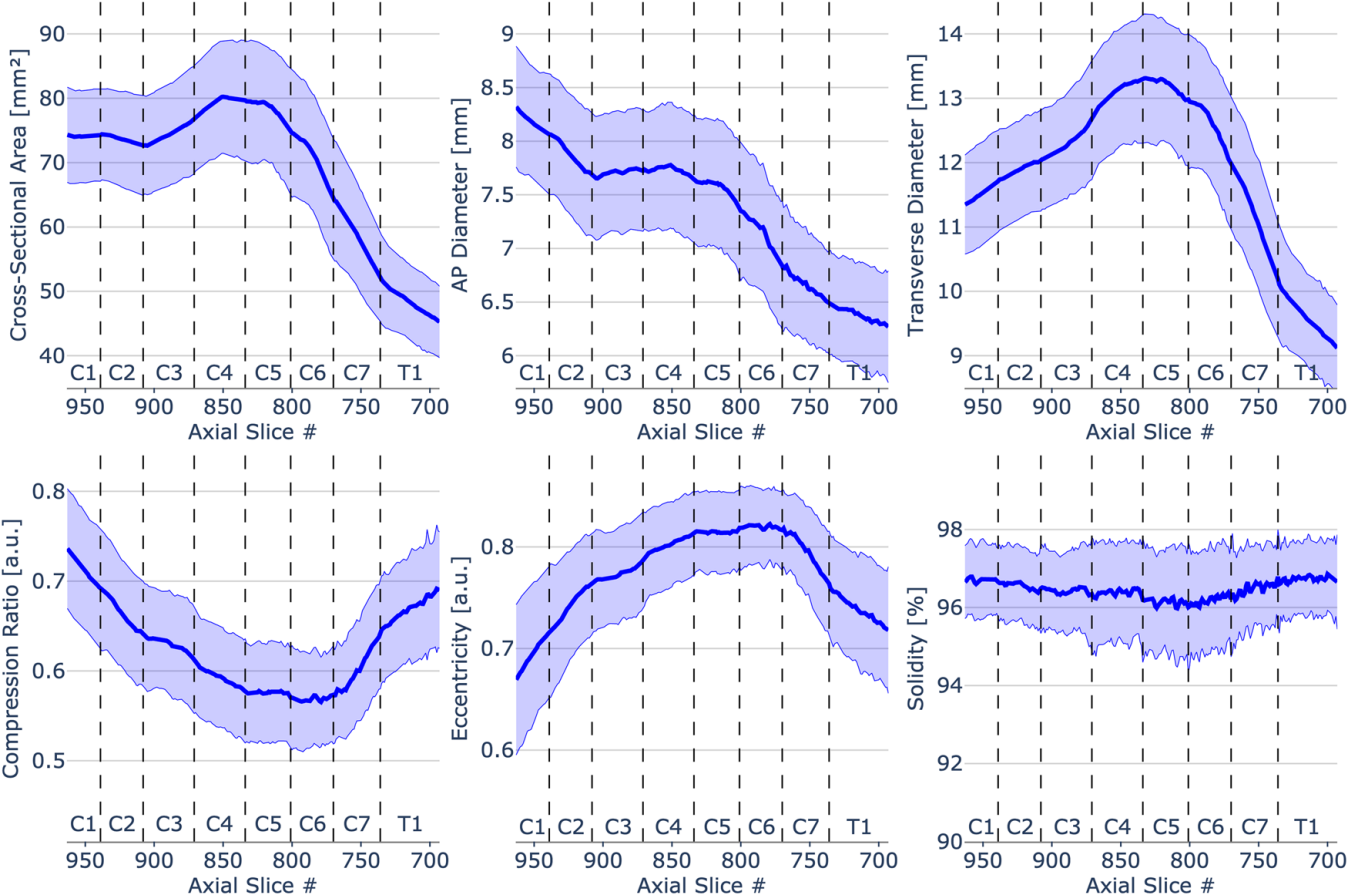
Spinal cord morphometric measures of 203 healthy participants across slices from C1 to T1 vertebral level. To see the interactive figure: https://preprint.neurolibre.org/10.55458/neurolibre.00017/.

Figure 2 shows morphometric measures plotted across individual slices with vertebral levels identified on the plot. Values were calculated as mean and standard deviation across 203 participants for each slice in the PAM50 space.

**Fig. 3.**
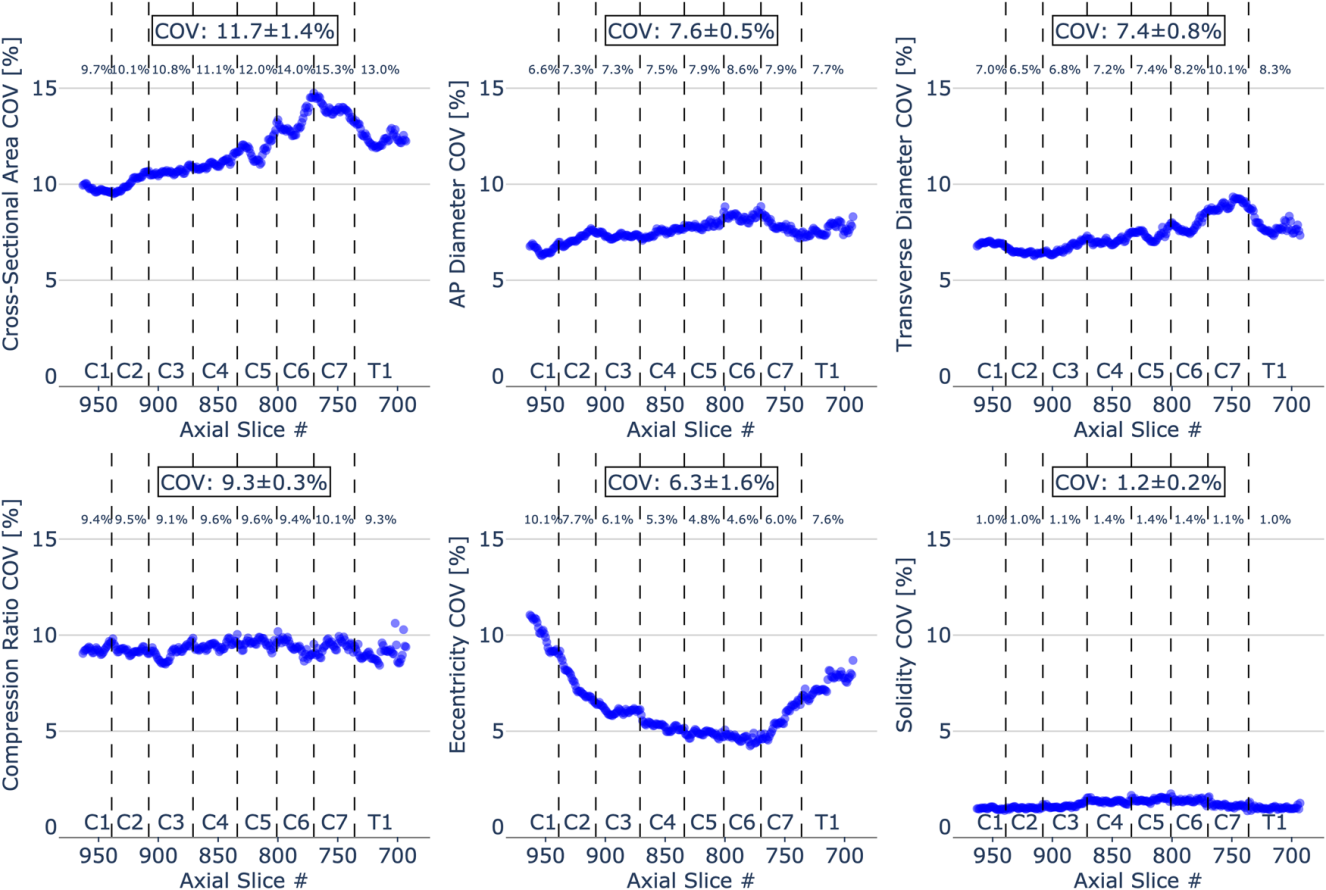
Coefficient of variation (COV) for individual morphometrics measures of 203 healthy participants across slices from C1 to T1 vertebral level. The mean COV for each vertebral level and the mean and standard deviation COV averaged across all slices are also shown. To see the interactive figure: https://preprint.neurolibre.org/10.55458/neurolibre.00017/.

Figure 3 shows a scatterplot for COV for each morphometric measure plotted across individual slices with vertebral levels identified on the plot. The figure also shows the mean COV for each vertebral level and the mean and standard deviation COV averaged across all slices. CSA demonstrated the highest mean COV of 11.7 ± 1.4%; on the other hand, solidity presented the lowest mean COV of 1.2 ± 0.2%.

### 3.2 Morphometric measures - influence of sex

**Fig. 4.**
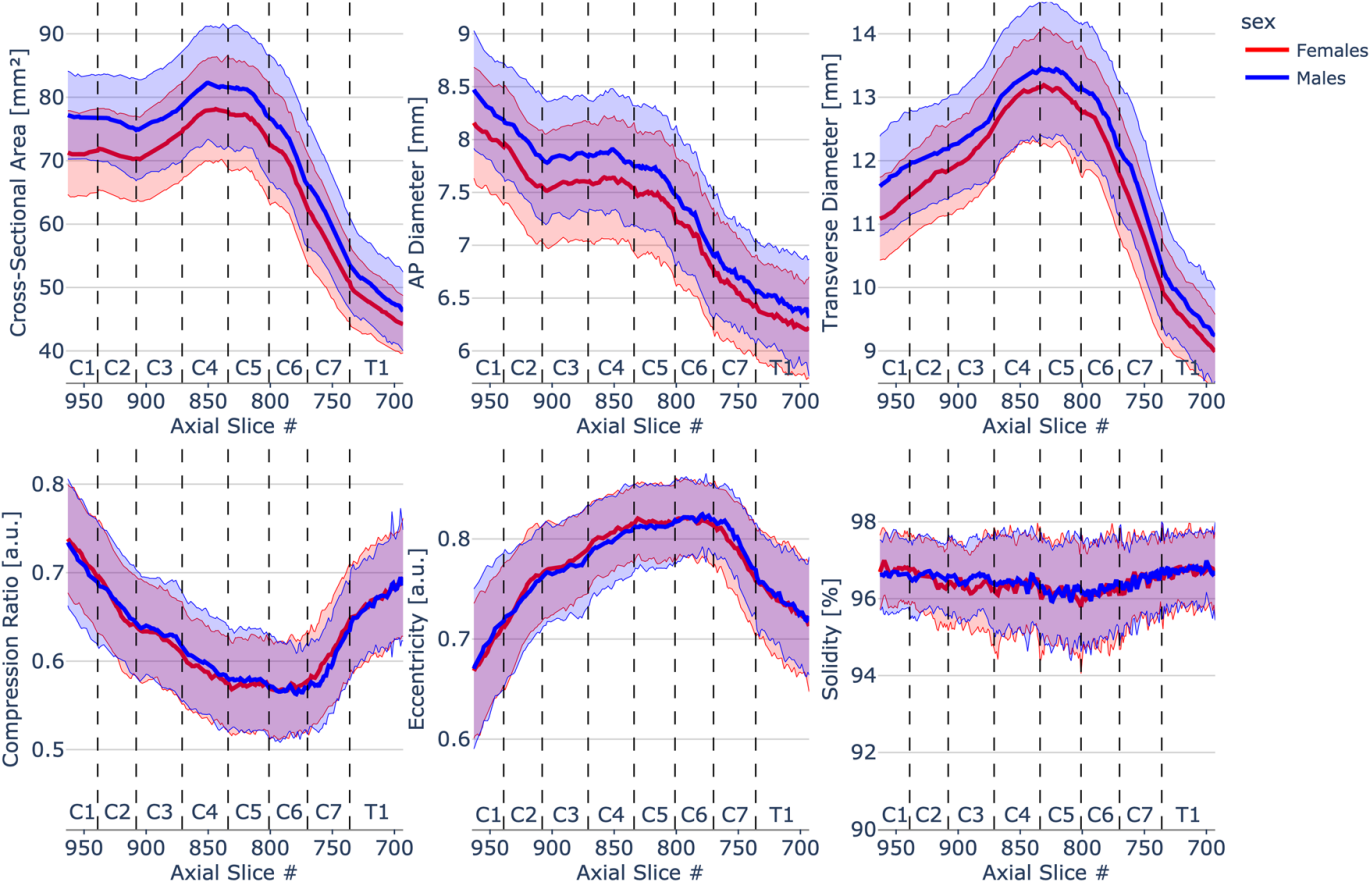
Spinal cord morphometrics measures of 203 healthy participants separated per sex (males: N = 105 and females: N = 98) across slices from C1 to T1 vertebral level. To see the interactive figure: https://preprint.neurolibre.org/10.55458/neurolibre.00017/.

Figure 4 presents morphometric measures plotted across individual slices with vertebral levels identified on the plot separated per sex (males: N = 105 and females: N = 98). Values were calculated as mean and standard deviation across 203 participants for each slice in PAM50 space. Wilcoxon rank-sum test showed significant differences (p < .001) between males and females for CSA, AP diameter and transverse diameter.

### 3.3 Morphometric measures - influence of MRI vendor

**Fig. 5.**
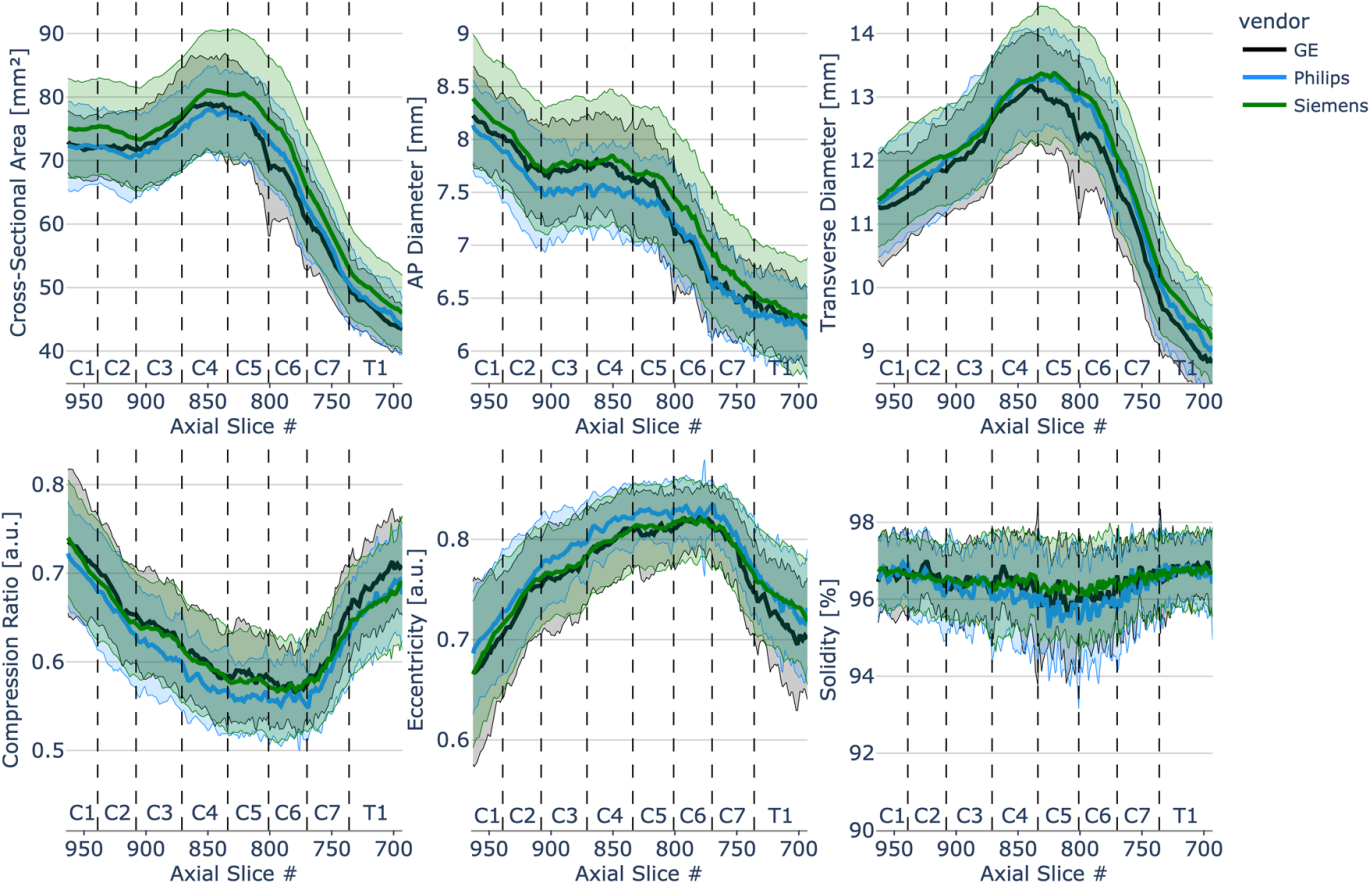
Spinal cord morphometrics measures of 203 healthy participants separated per MRI vendor (GE: N = 28, Philips: N = 36, and Siemens: N = 139) across slices from C1 to T1 vertebral level. To see the interactive figure: https://preprint.neurolibre.org/10.55458/neurolibre.00017/.

Figure 5 shows morphometric measures plotted across individual slices with vertebral levels identified on the plot separated for three MRI vendors (GE: N = 28, Philips: N = 36, and Siemens: N = 139). Values were calculated as mean and standard deviation across 203 participants for each slice in the PAM50 space. Wilcoxon rank-sum test showed significant differences (p < .001) for the following comparisons: Siemens vs. Philips and Siemens vs. GE for CSA and AP diameter, Siemens vs. Philips for transverse diameter, and Siemens vs. Philips and Philips vs. GE for compression ratio, eccentricity, and solidity.

### 3.4 Morphometric measures - influence of age

**Fig. 6.**
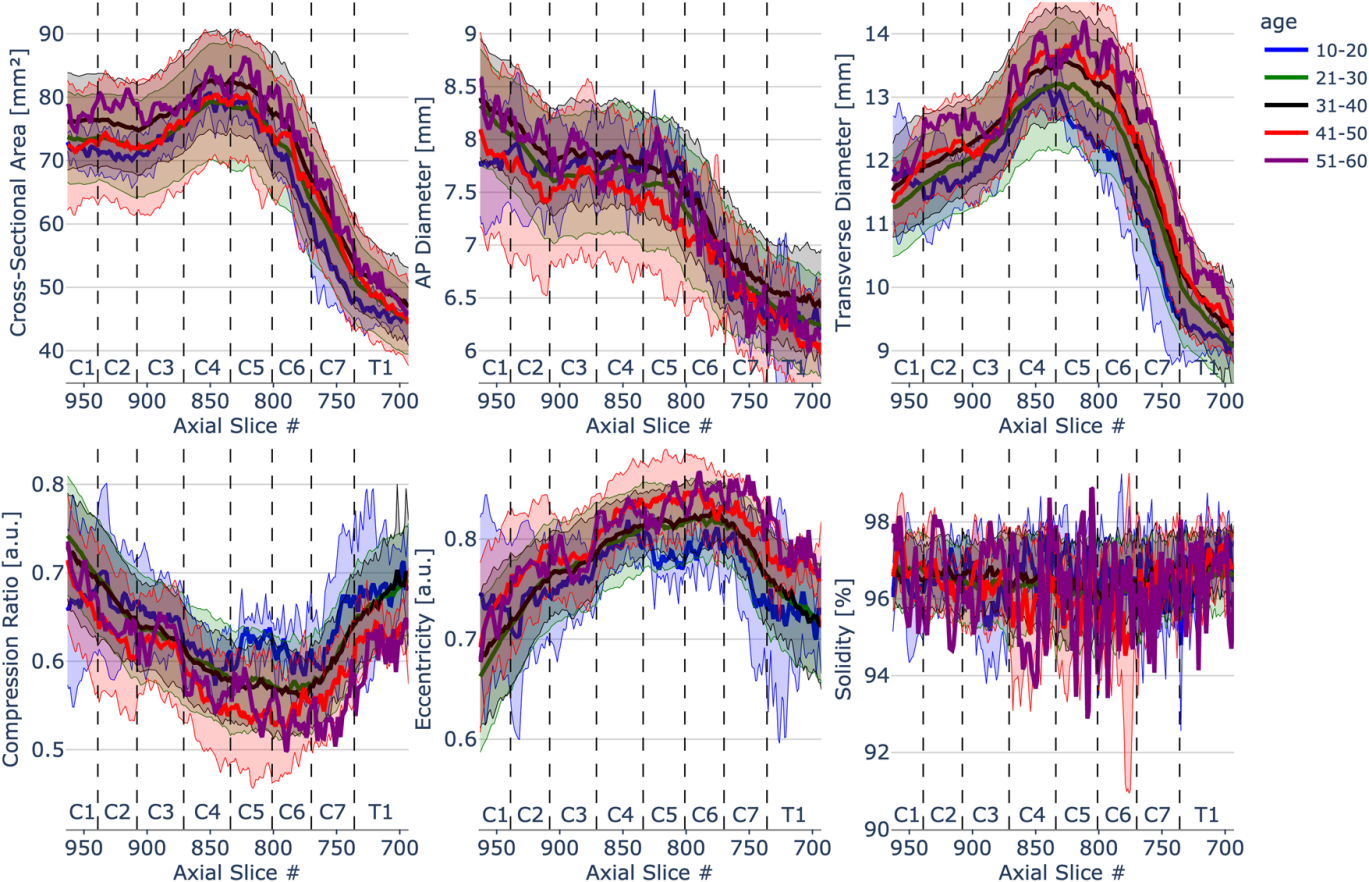
Spinal cord morphometrics measures of 203 healthy participants separated into five different age groups (10–20 decade: N = 4, 21–30 decade: N = 135, 31–40 decade: N = 53, 41–50 decade: N = 8, and 51–60 decade: N = 1) across slices from C1 to T1 vertebral level. To see the interactive figure: https://preprint.neurolibre.org/10.55458/neurolibre.00017/.

Figure 6 depicts morphometric measures plotted across individual slices with vertebral levels identified on the plot separated into five age decades. Values were calculated as mean and standard deviation across 203 participants for each slice in PAM50 space. Note that the groups are highly unbalanced (10–20 decade: N = 4, 21–30 decade: N = 135, 31–40 decade: N = 53, 41–50 decade: N = 8, and 51–60 decade: N = 1). For this reason, we did not perform the statistical comparison among the age decades. The interactive figure serves as a framework for adding better-distributed data in the future for a more informative exploration of the impact of age on spinal cord morphometrics.

### 3.5 Normative values

The normalized morphometric measures saved as comma-separated value (CSV) files (one file per subject) are accessible on GitHub (https://github.com/spinalcordtoolbox/PAM50-normalized-metrics/tree/r20230904)

Table 2 shows normative values for the whole cohort calculated as the mean and standard deviation across 203 subjects. Values are shown for slices in PAM50 space corresponding to the middle of each vertebral level and each intervertebral disc.

**Table 2.** Normative values of 203 healthy participants.

	Axial slice #	CSA [mm²]	AP diameter [mm]	Transverse diameter [mm]	Compression ratio [a.u.]	Eccentricity [a.u.]	Solidity [%]
Level C1	951	73.99 ± 7.12	8.17 ± 0.52	11.51 ± 0.8	0.71 ± 0.07	0.69 ± 0.07	96.73 ± 1.03
Disc C1-C2	939	74.27 ± 7.16	8.06 ± 0.56	11.71 ± 0.79	0.69 ± 0.07	0.72 ± 0.06	96.66 ± 0.92
Level C2	923	73.59 ± 7.41	7.86 ± 0.56	11.9 ± 0.76	0.66 ± 0.06	0.74 ± 0.05	96.65 ± 0.93
Disc C2-C3	908	72.67 ± 7.76	7.69 ± 0.57	12.02 ± 0.78	0.64 ± 0.06	0.76 ± 0.05	96.49 ± 1.15
Level C3	889	74.32 ± 7.91	7.72 ± 0.56	12.26 ± 0.82	0.63 ± 0.06	0.77 ± 0.05	96.35 ± 1.08
Disc C3-C4	871	76.61 ± 8.35	7.72 ± 0.56	12.66 ± 0.93	0.61 ± 0.06	0.79 ± 0.05	96.34 ± 1.47
Level C4	852	80.04 ± 8.8	7.77 ± 0.59	13.14 ± 0.91	0.59 ± 0.06	0.8 ± 0.04	96.44 ± 1.24
Disc C4-C5	834	79.68 ± 9.32	7.65 ± 0.6	13.3 ± 1.0	0.58 ± 0.06	0.81 ± 0.04	96.19 ± 1.6
Level C5	817	78.94 ± 8.91	7.61 ± 0.6	13.24 ± 0.93	0.58 ± 0.06	0.81 ± 0.04	96.22 ± 1.32
Disc C5-C6	801	74.92 ± 9.9	7.37 ± 0.63	12.98 ± 1.03	0.57 ± 0.05	0.82 ± 0.04	96.01 ± 1.68
Level C6	785	71.64 ± 9.0	7.2 ± 0.59	12.67 ± 0.94	0.57 ± 0.05	0.82 ± 0.04	96.26 ± 1.44
Disc C6-C7	770	64.45 ± 9.5	6.84 ± 0.6	11.98 ± 1.04	0.57 ± 0.05	0.82 ± 0.04	96.22 ± 1.48
Level C7	753	58.98 ± 8.12	6.68 ± 0.52	11.19 ± 0.98	0.6 ± 0.06	0.8 ± 0.04	96.59 ± 0.99
Disc C7-T1	736	52.25 ± 6.9	6.49 ± 0.49	10.19 ± 0.89	0.64 ± 0.06	0.76 ± 0.05	96.79 ± 1.21
Level T1	714	48.18 ± 5.81	6.38 ± 0.5	9.54 ± 0.71	0.67 ± 0.06	0.74 ± 0.06	96.83 ± 1.06

Values are shown as mean and standard deviation for slices in PAM50 space corresponding to the middle of each vertebral level and each intervertebral disc.

Tables 3 and 4 show normative values separated for sex calculated as the mean and standard deviation for 105 males and 98 females. Values are shown for slices in PAM50 space corresponding to the middle of each vertebral level and each intervertebral disc.

**Table 3.** Normative values of 105 healthy males.

	Axial slice #	CSA [mm²]	AP diameter [mm]	Transverse diameter [mm]	Compression ratio [a.u.]	Eccentricity [a.u.]	Solidity [%]
Level C1	951	76.78 ± 6.52	8.3 ± 0.49	11.77 ± 0.85	0.71 ± 0.07	0.7 ± 0.08	96.69 ± 1.1
Disc C1-C2	939	76.78 ± 6.77	8.18 ± 0.55	11.94 ± 0.82	0.69 ± 0.07	0.72 ± 0.07	96.58 ± 0.94
Level C2	923	76.19 ± 7.28	8.02 ± 0.56	12.09 ± 0.77	0.67 ± 0.06	0.74 ± 0.05	96.72 ± 0.9
Disc C2-C3	908	74.84 ± 8.03	7.81 ± 0.59	12.19 ± 0.8	0.64 ± 0.06	0.76 ± 0.05	96.6 ± 1.09
Level C3	889	76.41 ± 8.09	7.84 ± 0.57	12.42 ± 0.87	0.63 ± 0.06	0.77 ± 0.05	96.54 ± 1.04
Disc C3-C4	871	78.53 ± 8.62	7.84 ± 0.53	12.79 ± 1.02	0.62 ± 0.06	0.78 ± 0.05	96.42 ± 1.34
Level C4	852	82.06 ± 9.15	7.91 ± 0.58	13.25 ± 1.02	0.6 ± 0.06	0.8 ± 0.05	96.52 ± 1.33
Disc C4-C5	834	81.54 ± 9.28	7.75 ± 0.57	13.44 ± 1.07	0.58 ± 0.06	0.81 ± 0.04	96.26 ± 1.43
Level C5	817	81.07 ± 9.03	7.74 ± 0.58	13.38 ± 1.0	0.58 ± 0.06	0.81 ± 0.04	96.11 ± 1.3
Disc C5-C6	801	76.85 ± 9.91	7.47 ± 0.64	13.16 ± 1.01	0.57 ± 0.06	0.82 ± 0.04	96.21 ± 1.6
Level C6	785	73.52 ± 9.54	7.3 ± 0.59	12.83 ± 0.98	0.57 ± 0.05	0.82 ± 0.04	96.38 ± 1.34
Disc C6-C7	770	66.19 ± 9.86	6.9 ± 0.62	12.17 ± 1.06	0.57 ± 0.05	0.82 ± 0.04	96.34 ± 1.54
Level C7	753	61.03 ± 8.28	6.76 ± 0.51	11.43 ± 1.0	0.59 ± 0.05	0.8 ± 0.04	96.58 ± 1.04
Disc C7-T1	736	53.57 ± 7.23	6.54 ± 0.5	10.36 ± 0.89	0.63 ± 0.06	0.77 ± 0.05	96.82 ± 1.19
Level T1	714	49.46 ± 6.12	6.47 ± 0.5	9.66 ± 0.72	0.67 ± 0.06	0.74 ± 0.05	96.83 ± 0.98

Values are shown as mean and standard deviation for slices in PAM50 space corresponding to the middle of each vertebral level and each intervertebral disc.

**Table 4.** Normative values of 98 healthy females.

	Axial slice #	CSA [mm²]	AP diameter [mm]	Transverse diameter [mm]	Compression ratio [a.u.]	Eccentricity [a.u.]	Solidity [%]
Level C1	951	71.01 ± 6.52	8.03 ± 0.52	11.23 ± 0.65	0.72 ± 0.06	0.69 ± 0.06	96.76 ± 0.95
Disc C1-C2	939	71.59 ± 6.6	7.93 ± 0.55	11.47 ± 0.67	0.69 ± 0.06	0.71 ± 0.06	96.74 ± 0.9
Level C2	923	70.81 ± 6.51	7.7 ± 0.51	11.69 ± 0.68	0.66 ± 0.06	0.75 ± 0.05	96.58 ± 0.96
Disc C2-C3	908	70.34 ± 6.77	7.56 ± 0.53	11.85 ± 0.71	0.64 ± 0.06	0.76 ± 0.05	96.38 ± 1.21
Level C3	889	72.09 ± 7.08	7.6 ± 0.53	12.09 ± 0.74	0.63 ± 0.06	0.77 ± 0.05	96.14 ± 1.09
Disc C3-C4	871	74.56 ± 7.57	7.6 ± 0.57	12.52 ± 0.8	0.61 ± 0.06	0.79 ± 0.04	96.26 ± 1.6
Level C4	852	77.88 ± 7.91	7.63 ± 0.57	13.02 ± 0.76	0.59 ± 0.05	0.81 ± 0.04	96.36 ± 1.13
Disc C4-C5	834	77.69 ± 8.99	7.54 ± 0.62	13.15 ± 0.9	0.58 ± 0.06	0.81 ± 0.04	96.12 ± 1.76
Level C5	817	76.65 ± 8.22	7.47 ± 0.6	13.09 ± 0.83	0.57 ± 0.05	0.82 ± 0.04	96.33 ± 1.34
Disc C5-C6	801	72.86 ± 9.51	7.27 ± 0.61	12.79 ± 1.03	0.57 ± 0.05	0.82 ± 0.04	95.79 ± 1.74
Level C6	785	69.61 ± 7.93	7.09 ± 0.57	12.51 ± 0.86	0.57 ± 0.06	0.82 ± 0.04	96.13 ± 1.54
Disc C6-C7	770	62.58 ± 8.78	6.77 ± 0.59	11.77 ± 0.99	0.58 ± 0.06	0.81 ± 0.04	96.08 ± 1.41
Level C7	753	56.78 ± 7.36	6.6 ± 0.53	10.92 ± 0.88	0.61 ± 0.06	0.79 ± 0.05	96.6 ± 0.94
Disc C7-T1	736	50.84 ± 6.26	6.44 ± 0.47	10.02 ± 0.86	0.65 ± 0.06	0.76 ± 0.06	96.76 ± 1.23
Level T1	714	46.82 ± 5.14	6.28 ± 0.48	9.41 ± 0.68	0.67 ± 0.07	0.74 ± 0.06	96.82 ± 1.14

Values are shown as mean and standard deviation for slices in PAM50 space corresponding to the middle of each vertebral level and each intervertebral disc.

## 4 Discussion

This study introduced a framework to automatically normalize spinal cord morphometric measures and computed normative metrics from a public database of healthy adults. Normative values were reported in the PAM50 template reference space, which facilitates the comparison of results across past and future studies. Metrics were presented as interactive figures, allowing researchers to conveniently explore morphometric values and filter them according to sex, age, and MRI vendor. In this discussion, we address the demographics of the study used to generate the normative values, we discuss the technical details about bringing subject-wise measures to the PAM50 template, we compare the obtained measures with the literature, we provide recommendations for the application of these normative values in future research, and we address limitations and discuss future work.

### 4.1 Participants

Given our objective of constructing a database containing healthy normative morphometric values, two experienced radiologists evaluated MRI scans with a focus on the presence of spinal cord compression. The assessment revealed mild spinal cord compression in 24% of volunteers. This is lower compared to previous studies that have reported the prevalence of asymptomatic spinal cord compression in up to 40% of the otherwise healthy population (Kovalova et al., 2016; Smith et al., 2021). The lower number of participants with mild cord compressions in our study can be attributed to the lower age of our population (mean ± std = 28.7 ± 5.6; median = 28.0, range = 19–52) while Kovalova et al. reported a median age of 66 years ranging from 40 to 80 years. Additionally, Smith et al. reported that the prevalence of spinal cord compressions was significantly higher in participants >60 years old.

### 4.2 Normalization to PAM50 anatomical dimensions

The proposed normalization approach linearly interpolates the morphometric measures to the PAM50 anatomical dimensions based on the number of slices for each vertebral level in the native space and the PAM50 template. Compared to classical voxel-based morphometry performed on a template space, wherein the metrics are corrected using the Jacobian of the non-linear warping field, here, we take the morphometrics computed on the subject’s native space, and we interpolate them so that their coordinates match that of the PAM50 space. One notable advantage of this approach is that the normalized metrics are not impeded by possible mis-registration. Indeed, the slight mis-registration between the subject’s spinal cord and the PAM50 spinal cord would result in an incorrect Jacobian matrix, and hence inaccurate morphometrics. Registration to the PAM50 template typically involves spinal cord straightening, vertebral alignment between the image and the template, and iterative slice-wise non-linear registration (De Leener et al., 2017). Each of these steps might change the spinal cord shape and contour (see a relevant issue on GitHub: https://github.com/sct-pipeline/dcm-metric-normalization/issues/3). Here, the normalization only relies on the more reliable positions of the intervertebral discs.

A corollary of the proposed normalization approach is that it provides a morphometric value for each axial slice of the PAM50 template, i.e., every 0.5 mm along the superior–inferior direction. In comparison with morphometrics aggregated across multiple levels (what is commonly done along C2-C3 to evaluate cord atrophy in multiple sclerosis patients), such precision could be relevant in conditions in which local cord morphometric measures are useful, e.g., to assess compression along a few mm of cord tissue.

### 4.3 Morphometric measures

In this section, we discuss the notable morphometrics findings of our study and compare them with existing literature.

The increase in CSA around vertebral levels C4-C5 indicates the location of cervical enlargement, the source of the large spinal nerves that supply the upper limbs. This finding is consistent with anatomical textbooks (Standring, 2020), and previous in vivo (De Leener et al., 2018; Frostell et al., 2016; Horáková et al., 2022; Martin et al., 2017b; Mina et al., 2021; Rocca et al., 2019) and ex vivo (Calabrese et al., 2018; Kameyama, Hashizume, & Sobue 1996; Ko et al., 2004) studies. After the cervical enlargement (i.e., below level C5), the cervical spinal cord becomes smaller, which is mirrored by the decrease in CSA, AP diameter, and transverse diameter. The decrease in AP diameter along the superior–inferior direction, along with the changing trends in compression ratio and eccentricity, corresponds to the fact that the spinal cord is not cylindrical but rather changes its shape across levels from circular shape at C1 and C2 levels to a more elliptical shape around levels C5 and C6 (Standring, 2020).

Our results showed that females have smaller CSA, AP diameter, and transverse diameter relative to males across all vertebral levels. This has been previously reported (Bédard & Cohen-Adad, 2022; Engl et al., 2013; Papinutto et al., 2020; Rashid et al., 2006; Solstrand Dahlberg, Viessmann, & Linnman, 2020; Yanase et al., 2006).

Inter-subject variability of morphometric measures is presented in Figure 3. We noted that the CSA has the highest variability (mean COV of 11.7 ± 1.4%), while the solidity has the lowest (mean COV of 1.2 ± 0.2%). In comparison, Bédard et Cohen-Adad reported a 9.96% COV for CSA measured on T1-w images at the C2-C3 level (Bédard & Cohen-Adad, 2022). Variation in COV at lower cervical levels (around intervertebral discs C4/C5, C5/C6, and C6/C7) apparent in CSA, AP diameter, transverse diameter, and solidity (Fig. 3), might be attributed to mild anatomy alterations such as a disc protrusion. Although the disc protrusions primarily affect the spinal canal, they might also cause slight spinal cord anatomy variation reflected by higher COV for some morphometric measures. It is important to note that differences in COV across all cord morphometrics are relevant if, for example, two metrics are particularly sensitive to a given pathology, but one shows less inter-subject variability. While CSA and AP diameter are popular metrics, others might be equal or more relevant, given their lower inter-subject COV. Future studies are needed to identify other relevant morphometric markers across various diseases.

Hereafter, we compare the observed morphometric measures with those from the literature. De Leener et al., 2018; performed a study in 50 healthy subjects, where they reported a CSA of 77.46 ± 8.45 mm^2^ at the level of C3/C4 intervertebral disc compared to 76.61 ± 8.35 mm^2^ in our study. That study used a similar T2-w sequence (except for the resolution: 1 mm in their study vs. 0.8 mm in ours) and a similar cord segmentation method (PropSeg vs. *sct_deepseg_sc* trained on PropSeg ground truths), hence the excellent level of agreement was expected. In contrast, other studies reported either smaller or larger CSA. For instance, (Horáková et al., 2022) obtained a CSA of 71.7 ± 8.2 mm^2^ at the C3/C4 intervertebral disc, while (Kesenheimer et al., 2021) measured a CSA of 87.4 ± 8.31 mm^2^. Another study based on the UK Biobank database (N = 804) measured a CSA of 66.4 ± 6.61 mm^2^ at C2-C3 vertebral levels (Bédard & Cohen-Adad, 2022). Here, we measured larger CSAs: 73.59 ± 7.41 mm^2^ at C2 and 74.32 ± 7.91 mm^2^ at C3 vertebral levels. These differences in CSA can be attributed to various factors, including variations in population ages, variations in the segmentation methods used, and differences in MRI sequence parameters and/or reconstruction filters. For example, T2-w scans generally yield larger CSA compared to T1-w scans (Cohen-Adad et al., 2021b), as further discussed in 4.5. Limitations and future work.

As for AP diameter and transverse diameter, values measured in this study correspond with population estimates from Frostell et al. (2016). For instance, we measured an AP diameter of 7.86 ± 0.56 mm and a transverse diameter of 11.9 ± 0.76 mm for the C2 vertebral level, while (Frostell et al., 2016) reported an AP diameter of 7.9 ± 1.6 mm and a transverse diameter of 12.3 ± 2.4 mm for the same vertebral level.

Regarding ex vivo studies, a study of 13 male and 2 female adult cadavers reported CSA of ~71 mm^2^ and AP diameter of ~12 mm at the C3 level (Ko et al., 2004). Another study of 5 male and 7 female adult cadavers reported smaller values of CSA of 52.6 ± 5.2 mm^2^ and AP diameter of 10.7 ± 0.7 mm at the C3 level (Kameyama, Hashizume, & Sobue, 1996). While these quantitative reports on cadaveric populations are informative, it is difficult to compare those with in vivo MRI-based measures due to the inherent characteristics of ex vivo measures, such as tissue shrinking due to post-fixation, and the altered shape of the excised spinal cord without the surrounding cerebrospinal fluid and dura.

### 4.4 Using the normative database in future studies

The proposed normalization approach is integrated into the *sct_process_segmentation* function (with the new flag “-normalize-PAM50” as of SCT v6.0). This new feature requires only the subject’s spinal cord segmentation and disc levels as inputs, and it outputs the subject’s morphometrics within the PAM50 anatomical dimensions. Bringing multiple subjects to the same PAM50 dimensions is a convenient means to perform group statistics and compare an individual subject with the healthy normative database (e.g., based on a computed Z-score). As previously mentioned, group studies can be restricted to given demographic and biological factors (e.g., age, sex, and height) and acquisition properties (e.g., image contrast and MRI vendor) as long as these data are available in the given population. Using BIDS’ (Gorgolewski et al., 2016) participants.tsv file to encode such information is recommended. This filtering feature, e.g., for sex, is relevant and commonly used to normalize spinal cord morphometric measures (Bédard & Cohen-Adad, 2022; Kesenheimer et al., 2021; Mina et al., 2021; Papinutto et al., 2020; Rashid et al., 2006; Rocca et al., 2019).

The proposed methodology supports the normalization of morphometric values for all six metrics simultaneously, offering a convenient way to explore the relationship between individual morphometric measures. This could be particularly useful for assessing changes in spinal anatomy between levels or for identifying levels of spinal cord compression. Indeed, the normalization of compression metrics, utilizing our healthy normative database, has been added to the *sct_compute_compression* function and is currently being evaluated in cohorts of traumatic and non-traumatic spinal cord injury patients.

### 4.5 Limitations and future work

The T2-w images from the open-access *spine-generic* dataset only cover the spinal cord from C1 to about T1 and have a relatively narrow age range (with 93.6% of subjects aged 21–40 years). Also, the data come predominantly from Siemens scanners, which skews the morphometric values towards a particular vendor. Despite these limitations, it remains the largest open-access database of multi-contrast spinal cord MRI data. Future studies should focus on adding datasets covering the whole spinal cord, acquired using other manufacturers, and with more heterogeneous age distributions.

The morphometric measures were derived solely from T2-w MRI contrast and using a segmentation method trained specifically for this contrast (Gros et al., 2019). As reported previously, morphometric measures are strongly dependent on the image contrast and on the segmentation method (Bédard et al., 2023; Cohen-Adad et al., 2021b; Kim et al., 2015; Weeda et al., 2019). For example, a previous study comparing SCT’s *sct_deepseg_sc* algorithm on T2-w and T1-w contrasts showed that CSA was overall higher on T2-w vs. T1-w for all three major MRI manufacturers (Cohen-Adad et al., 2021b). Another study showed discrepancies between automatic and semi-automatic segmentation algorithms for the same contrast (Weeda et al., 2019). Therefore, in this study, we decided to only consider one contrast (T2-w) and one segmentation method (*sct_deepseg_sc*), anticipating future efforts in normalizing imaging protocols (Cohen-Adad et al., 2021a) and in making segmentation algorithms less sensitive to image contrast (Bédard et al., 2023).

Future directions will focus on validating the proposed methods in pathologies such as traumatic and non-traumatic spinal cord injury and multiple sclerosis. This validation process will provide valuable insights into the applicability and accuracy of the methods in the context of various spinal cord conditions.

## 5 Conclusion

We introduced a new approach for the normalization of spinal cord morphometric measures using the PAM50 spinal cord template. We built an interactive database of spinal cord morphometric values across 203 healthy adults. The database can be used to normalize spinal cord morphometric features, stratified according to factors such as sex, age, and MRI vendors. This database can also be used to further inspect demographic, biological, and image acquisition factors associated with inter-subject variability.

The proposed methodology and results are open-source and fully reproducible. The database and normalization method are applicable to new datasets via the Spinal Cord Toolbox (SCT) v6.0 and higher.

## Data Availability

The anonymized and defaced *spine-generic* dataset is organized according to the BIDS standard and managed using git-annex in the following GitHub repository: https://github.com/spine-generic/data-multi-subject/tree/r20230223. The processing used to compute morphometric measures of individual subjects in the PAM50 template space is available at: https://github.com/sct-pipeline/dcm-metric-normalization/blob/r20230222/scripts/process_data_spine-generic.sh. The computed morphometric measures in CSV format are available at: https://github.com/spinalcordtoolbox/PAM50-normalized-metrics/tree/r20230904. The normalization method is available through the *sct_process_segmentation* function as part of the Spinal Cord Toolbox (SCT) v6.0 and higher: https://github.com/spinalcordtoolbox/spinalcordtoolbox/tree/6.0. The interactive figures in this article were produced using the NeuroLibre publication workflow powered by Plotly (https://plotly.com) and are available at: https://preprint.neurolibre.org/10.55458/neurolibre.00017/.
